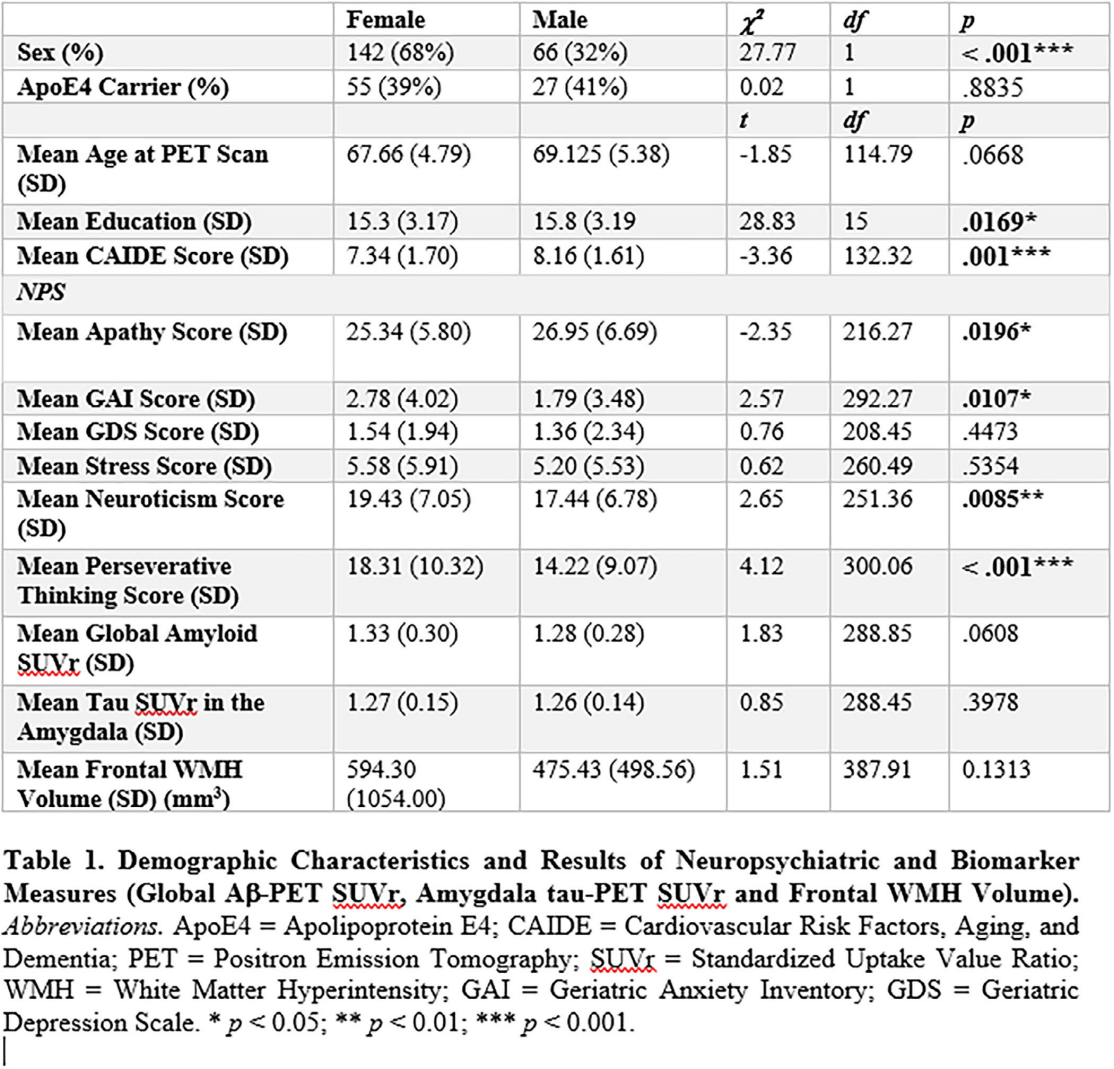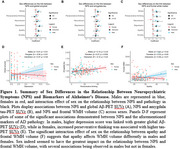# Sex‐specific effects of neuropsychiatric symptoms on amyloid and tau pathology and white matter hyperintensity volume in preclinical Alzheimer's disease

**DOI:** 10.1002/alz70857_103725

**Published:** 2025-12-24

**Authors:** Aurélie Garrone, Yara Yakoub, Alfonso Fajardo, Ting Qiu, Amelie Metz, Mahsa Dadar, Valentin Ourry, Sylvia Villeneuve, PREVENT‐AD Research Group

**Affiliations:** ^1^ Douglas Mental Health University Institute, Centre for Studies on the Prevention of Alzheimer's Disease (StoP‐AD), Montréal, QC, Canada; ^2^ Integrated Program in Neurosciences, McGill University, Montréal, QC, Canada; ^3^ Department of Psychiatry, McGill University, Montréal, QC, Canada; ^4^ Douglas Mental Health University Institute, Montréal, QC, Canada; ^5^ Douglas Mental Health University Institute, Montreal, QC, Canada

## Abstract

**Background:**

Neuropsychiatric symptoms (NPS) are highly prevalent in Alzheimer's disease (AD) and may constitute both risk factors and early signs of the disease. Cross‐sectional and longitudinal studies have shown that NPS are associated with increased Aβ and tau burden in preclinical AD populations. The amygdala, a brain region involved in emotion modulation and processing, is among the most vulnerable regions to exhibit early tau deposition. Further, white matter hyperintensity (WMH) volume, another neurological correlate of AD, has been linked to worsening NPS, especially in frontotemporal regions. Interestingly, sex may also affect the incidence and prevalence of NPS in AD, with females being at higher risk than males. We assessed the associations between NPS and 1) Aβ, 2) amygdala tau and 3) frontal WMH burden in cognitively unimpaired older adults. Given that females reportedly exhibit greater NPS prevalence and severity, we hypothesized that these associations would be stronger in females; at higher levels of NPS, females should therefore exhibit greater pathology.

**Method:**

208 participants from the longitudinal PREVENT‐AD cohort received amyloid (^18^F‐NAV4694) and tau (^18^F‐Flortaucipir) PET‐scans. We conducted linear regressions using sex as an interaction term to assess the effect of NPS (apathy, anxiety, depression, stress, perseverative thinking and neuroticism) on global Aβ‐PET standard uptake values ratio (SUVr), tau‐PET SUVr in the amygdala, and WMH volume in the frontal lobe. These associations were also examined separately in males and females. All models were corrected for age, years of education, APOE4 carrier status and cardiovascular risk.

**Result:**

Despite females exhibiting greater NPS, several associations, particularly with WMH volume, were found in males, but not in females. Stress and depression were also associated with amyloid and amygdala tau deposition in males, while perseverative thinking was associated with amygdala tau deposition in females.

**Conclusion:**

We found associations between NPS and both MRI and PET markers of AD in cognitively unimpaired individuals at risk of AD. Some of these associations were stronger in males, despite females being at increased risk of AD and known to exhibit a higher prevalence of NPS.